# Books and Authors

**Published:** 1905-08

**Authors:** 


**Operative Surgery.** By **Joseph D. Bryant,** M.D., Professor of the Principles and Practice of Surgery, Operative and Clinical Surgery, University and Bellevue Hospital Medical College. In two octavo volumes. Fourth edition. Vol. 1. General Principles, Anesthetics, Treatment of Operation Wounds, Operations on Veins, Amputations, Plastic Surgery, etc., etc. With 898 illustrations, 61 in colors. Vol. 2. Operations on the Viscera connected with the Peritoneum, and Miscellaneous Operations. With 895 illustrations, 39 in colors. New York and London: D. Appleton & Co. 1905. (Price, cloth $10.00.)

None but a practical surgeon could write such a treatise as this. It has the tone and color from cover to cover of a man skilled at the operating table and one accustomed to deal with whatever may present itself,—simple or complicated,—for surgical consideration.

This work, now in its fourth edition, is in effect a new treatise, having been reset and almost entirely rewritten, hence it represents the surgery of the immediate present, a fact that means a good deal to the undergraduate and the junior practitioner, not to mention, also, the more experienced physician who must needs have for reference the very latest and best authority.

The first volume begins with general considerations in which preparations for operations are dealt with, from the simplest to the most elaborate, and then the subject itself is taken up. The control of hemorrhage, the treatment of operation wounds, the ligature of arteries, appear in consecutive order, followed by the surgery of the several systems,—vascular, nervous, ligamentous, etc., and osseous systems. Amputations, always possessing fascinating interest for the novitiate, are elaborately presented. The experienced surgeon always endeavors to avoid amputation, but when it must needs be done is prepared always to do the work skilfully and conservatively. This is what Bryant teaches.

The surgery of deformities and plastic surgery are two topics that appeal to the ingenuity as well as mechanical dexterity of the surgeon, and are herein set forth in an attractive manner; so, too, are operations on the mouth, pharynx, esophagus, and neck, which latter group carries the volume up to the index.

The second volume takes up operations on the viscera connected with the peritoneum, embracing the surgery of the gastrointestinal tract, appendix, liver and gallbladder, kidney, ureters, spleen, pancreas, subphrenic abscess, and hernia—which are considered in much detail. This one chapter of 430 pages is probably the largest single chapter ever published in a medical book. Especially do we invite attention to the section of it relating to intestinal operations and hernia,—the most elaborate and satisfactory yet published in a work on general surgery.

Operations on the anus and rectum form the subject of a chapter of seventy pages, and is a comprehensive presentation of a perplexing, though very important surgical topic. The thorax, including excision of the breast, is the next region to receive operative attention; and this is followed by a chapter of seventy-four pages on operations relating to the urinary bladder. This, too, is a chapter deserving special commendation; it is replete with skilful technic and suggestive method.

The final chapter considers operations on the scrotum and penis, and miscellaneous operations, the latter embracing such as find no ready place in the general groups and regions, as adopted by the author in the arrangement of his treatise. Each volume contains a good index,—a consideration of no mean importance. Together the volumes contain 1,559 pages, yet there is scarcely a superfluous word; the sentences are terse, clear, and full of meaning; indeed, the entire text is an admirable example of English rhetoric put into compact form, yet full of expression. The illustrations are unusually striking and explanatory of the text and their number is astonishing.

We have aimed to call attention to some of the more important features of this great work, but not to review it in the ordinary sense of the work. This would scarcely be necessary in a treatise that has reached its fourth edition and is known wherever surgery is studied and taught.

**International Clinics.** A Quarterly of Illustrated Clinical Lectures and especially prepared articles on Treatment, Medicine, Surgery, Neurology, Pediatrics, Obstetrics, Gynecology, Orthopedics, Pathology, Dermatology, Ophthalmology, Otology, Rhinology, Laryngology, Hygiene and other topics of interest to students and practitioners. By leading members of the medical profession throughout the world. Edited by **A. O. J. Kelly,** A.M., M.D., Philadelphia. Volume I. Fifteenth series. 1905. Philadelphia and London: J. B. Lippincott Co. (Cloth, $2.00.)

The topics which this book deals with are: (1) treatment of cardiac asthma, by P. Merklea; (2) treatment of cirrhosis of the liver, unusual syphilis, tuberculosis, suffocative catarrh, and mucomembranous enterocolitis, by Albert Robin; (3) therapeutic indications in infected cholelithiasis, by A. Chauffard; (4) carbohydrates of human urine in health and in disease, by Carstairs Douglas; (5) the eye and the hand in the diagnosis of heart disease, by James J. Walsh; (6) early diagnosis of heart disease in children, by J. Porter Parkinson; (7) aortic stenosis, adherent pericardium, by Morris Manges; (8) intestinal adhesions, and hepotoptosis, by A. L. Benedict; (9) skin grafting in the late treatment of severe burns involving extensive areas of skin, by Archibald Young; (10) starvation of malignant growths by depriving them of blood supply, by Robert H. M. Dawbarn; (11) new operative method for the total extirpation of the larynx, by Francesco Durante; (12) treatment of knee-joint disease, by Russell A. Hibbs; (13) treatment of Glenard's disease, by A. Ernest Gallant; (14) morphinomania, cocomania, and general narcomaina, and their legal consequences, by Charles K. Mills; (15) a case of cerebellar tumor, by J. Walter Carr; (16) two cases of ocular palsy dependent on a lesion in the neighborhood of the sphenoidal fissure, by Edwin Bramwell; (17) anterior and posterior parietal presentations of the head in slightly flattened pelves.

Following these lectures is a résumé of the progress of medicine during 1904. The volume is a most interesting one, the contents varied, and the articles are well selected.

---

**A Textbook of Legal Medicine.** By **Frank Winthrop Draper,** A.M., M.D., Professor of Legal Medicine in Harvard University. Octavo, 573 pages, illustrated. Philadelphia, New York, London: W. B. Saunders & Co. 1905. (Cloth, $4.00 net.)

No doubt too little instruction on this subject, some of it of inferior quality, is imparted to undergraduate students. This book, if adhered to as a guide, will do much toward improving both quality and quantity of teaching on medical jurisprudence.

The chapter on medical evidence and the medical witness is at once brief and instructive. It does not contain too much advice to be practical, and it states the whole question tersely and adequately. If its precepts be studied and followed it will serve to keep the medical witness off the shoals and rocks of those intricate channels sometimes found in the seas of trial courts.

The chapters on criminal abortion,—there are two of them,—should be studied carefully by every physician in active practice, and especially by every junior. Abortion is a crime of too frequent occurrence to make it appear as an innocent violation of nature's physiology; those who perpetrate it seek to cover their tracks in the most subtle manner, and it often baffles the skill of a Sherlock Holmes to unravel the cunning network the guilty weave around them. Professor Draper deals with the subject from the cold standpoint of facts and not with the dilettanteism of the idealist.

Preceding the aforementioned topic the author devotes three chapters to the consideration of rape in its various aspects. No more revolting crime can come before the courts for trial than that which involves the ravishing of a young girl. No one ever feels sympathy for such a criminal, and, generally speaking, the full punishment of the law meets approval in such cases. But it must not be forgotten that a liability exists to blackmail, the temptation to extortion being all the greater when the dread of exposure is so to be feared. The preparation of a physician who may be called upon to examine a female who alleges rape must be complete. The accuser and the accused must each be examined if possible and all the evidence carefully weighed. Draper presents much material bearing on this important subject that cannot but aid in the solution of many difficult problems connected with it.

The book is full of excellent teachings on many other topics, which time and space forbid us so to mention. It is a treatise that should become a guide to the most accomplished expert medical jurist; it will afford him the latest thought concerning all the various features of medico-legal work, and properly equip him to meet the subtle cross-examination of the craftiest lawyer.

---

**The Pharmacopeia of the United States of America.** Eighth Decennial Revision. By Authority of the United States Pharmacopeial Convention held at Washington, D. C., 1900. Revised by the Committee of Revision and Published by the Board of Trustees. Official from September 1, 1905. P. Blakiston's Son & Co., Philadelphia, Agents.

The eighth revision of the pharmacopeia has been delayed in issue, but is none the less welcome; indeed, the appetite for the book has been sharpened by its tardiness. The chairman of the committee of revision, Professor Joseph P. Remington asks us to call attention to the change in the strength of tincture of aconite, tincture of veratrum, and tincture of strophanthus which will go into effect officially September 1, 1905.

The strength of tincture of aconite has been reduced from 35 per cent. to 10 per cent., and that of tincture of veratrum from 40 per cent. to 10 per cent. The strength of tincture of strophanthus has been increased from 5 per cent. to 10 per cent. These changes have been made in order to conform to the standards adopted by the International Conference on Potent Remedies, held at Brussels in September, 1902, the object being to make uniform the strength of potent remedies in all parts of the world.

The work entailed in every revision of the pharmacopeia is enormous and this one appears to have been more laborious than usual. It contains a vast amount of material and should meet the approbation of physicians, chemists, and druggists.

---

**American Edition of Nothnagel's Practice.** Diseases of the Blood (Anemia, Chlorosis, Leukemia, Pseudoleukemia). By **Dr. P. Ehrlich,** Frankfort a. Main; Dr. A. Lazarus, Charlottenburg; Dr. K. von Noorden, Frankfort a. Main; and Dr. Felix Pinus, Berlin. Edited, with additions, by Alfred Stengel, M.D., Professor of Clinical Medicine, University of Pennsylvania. Octavo volume of 714 pages, fully illustrated. Philadelphia and London: W. B. Saunders & Co. 1905. (Cloth, $5.00 net; half morocco, $6.00 net.)

Hematology has become an essential study in the curriculum of medicine, hence authoritative presentations of its more important scientific aspects, as detailed in this work, become of great value to every progressive physician. The anemias, chlorosis, and the leukemias are herein dealt with quite exhaustively, and the several articles represent the most advanced thought relating to each topic. The editor has added notes in brackets, especially in the course of the article on anemia. Ehrlich's teachings have met with opposition, and the contrary views are referred to here and there by Stengel, whose own work on the normal and pathologic histology of the blood has taken high place.

The recent announcement of the death of Nothnagel adds an interest of tragic sadness to this and the volumes of this series yet to issue.

---

**Bacteriology and Surgical Technic for Nurses.** By **Emily M. A. Stoney,** Superintendent of the Training School for Nurses, Saint Anthony's Hospital, Rock Island, Ill. Second edition, revised and enlarged by Frederic R. Griffith, M.D., New York. Duodecimo volume of 278 pages. Illustrated. Philadelphia, New York, London: W. B. Saunders & Co. 1905. (Cloth, $1.50 net.)

The reviser of this excellent treatise has found it necessary to make but few alterations in the original text. The chief changes in this edition are in the nature of additions; these include a few illustrations, a chapter on minor procedures, one dealing with obstetrical nursing, another on bandaging and dressings, and, finally, one concerning the nurse herself. A glossary appears at the end of the text and an index of excellence serves to complete a most useful book,—one of the very best of its kind.

---

**Transactions of the Medical Association of the State of Alabama.** Annual meeting held at Mobile, April 19-22, 1904. **Lewis C. Morris,** M.D., Secretary.

The book of transactions of this staunch veteran society are ever welcome. This volume contains the usual minutes of proceedings, orations, addresses, and scientific papers pertaining to the annual meeting of the association. This body has taken great interest for many years in questions pertaining to public health,—preventive medicine. A most interesting paper on pure drinking water for country homes and villages unequipped with water works was presented by Professor Edgar B. Kay, of the University of Alabama, who discussed the subject in the light of advanced engineering and sanitary science. The volume is full of good things and shows excellent discipline of the members.

---

**Progressive Medicine, Volume VII., June, 1905.** A Quarterly Digest of Advances, Discoveries and Improvements in the Medical and Surgical Sciences. Edited by **Hobart Amory Hare,** M.D., Professor of Therapeutics and Materia Medica in the Jefferson Medical College of Philadelphia. Octavo, 346 pages, illustrated. Philadelphia and New York: Lea Brothers & Co. (Per annum, in four cloth-bound volumes, $9.00; in paper binding, $6.00, carriage paid to any address.)

The contributors to this number of progressive medicine are William B. Coley whose subject is hernia; Edward Milton Foote, on the surgery of the abdomen (except hernia) ; John G. Clark, on the topic of gynecology; Alfred Stengel, who contributes articles on diseases of the blood, diathetic and metabolic diseases and on diseases of the spleen, thyroid gland, and lymphatic system; and Edward Jackson, who prepared the article on ophthalmology. It is one of the best numbers yet issued and contains some of the very latest methods and thought on these several topics.

---

**Studies in the Psychology of Sex—Sexual Selection in Man.** I. Touch. II. Smell. III. Hearing. IV. Vision. By **Havelock Ellis.** Pages xii.-270. Sold only by subscription to physicians, lawyers, and scientists. Philadelphia: F. A. Davis Company. 1905. (Cloth, $2.00 net.)

The continuation of these studies has evolved this somewhat bizarre series of essays. It is difficult to place the writings of this author, but they belong somewhere between the scientific and the sensual, between the useful and the libidinous, partaking sometimes of the one, and again of the other. Nevertheless, Ellis exhibits great industry and close study of the entire subject. The book will be read with interest by many persons. It would be well to restrict the circulation of such a book within the narrowest limits of scientific circles. An attempt is made to do this by the publisher and we hope with entire success.

